# Community-based infant hearing screening: Outcomes of a rural pilot programme

**DOI:** 10.4102/sajcd.v71i1.1045

**Published:** 2024-10-31

**Authors:** Khomotjo S. Kgare, Karin Joubert

**Affiliations:** 1 Department of Rehabilitative Sciences, Faculty of Health Sciences, University of Fort Hare, East London, South Africa; 2 Department of Audiology, Faculty of Humanities, University of the Witwatersrand, Johannesburg, South Africa

**Keywords:** Community-based, universal newborn hearing screening, developing context, rural

## Abstract

**Background:**

Community-based universal newborn hearing screening (UNHS) has not been fully realised in South Africa despite the availability of contextually relevant early hearing detection and intervention guidelines. Research has confirmed the feasibility of implementing UNHS programmes in urban contexts; however, limited information exists for rural contexts.

**Objectives:**

The aim of the study was to describe the outcomes in terms of coverage rate, referral rate and follow-up rate of a 1-year UNHS pilot programme implemented at three primary health care (PHC) clinics in the Limpopo province.

**Method:**

A descriptive retrospective review of 2 302 audiological records of infants who underwent NHS between July 2014 to June 2015 was conducted.

**Results:**

The mean age at first-stage screen was 112 days (16 weeks). The coverage rate was 87% for the infants screened at 3- and 10- days clinic visits and 27% for infants screened at the 6-week immunisation visit. The first-stage referral rate was 33.9% and 8.3% for the overall second stage referral for diagnostic audiology services. The follow-up rate for rescreens at the clinical level was 77%, while for initial diagnostic assessments, it was 26%.

**Conclusion:**

Although not all benchmarks were met within the first year of implementation, the high coverage- and low referral rates, especially in the last 6 months, are the first steps in improving the outcomes of the screening programme.

**Contribution:**

The findings confirm the feasibility of implementing community-based UNHS programmes in rural areas in South Africa. Regular monitoring and evaluation contribute to the success of screening programmes.

## Introduction

Newborn and infant hearing screening (NIHS) is a secondary prevention strategy that involves screening newborns and infants for potential hearing loss shortly after birth or during the early stages of development (Olusanya et al., 2014). The goal is to ensure that all infants have the opportunity to develop optimal communication skills and reach their full potential by detecting hearing loss as early as possible and providing timely and appropriate intervention. Research suggests that early intervention for hearing loss positively impacts speech and language development, cognitive development, social and emotional development as well as academic achievement (Maluleke et al., 2021). In the absence of other impairments, the speech and language skills of hearing-impaired children who received early intervention are on par with normal-hearing peers (Vohr et al., 2008).

Universal newborn hearing screening (UNHS) and targeted newborn hearing screening (TNHS), also referred to as at-risk screening, are two different approaches commonly used for NIHS (Kanji, 2021). Although these approaches are designed to identify hearing loss in infants, they target different populations and have distinct purposes. Targeted newborn hearing screening involves identifying and screening infants with one or more risk factors that increase their likelihood of hearing loss (Kanji, 2021). Universal newborn hearing screening involves screening all infants, regardless of the presence of any risk factors risk factors, shortly after birth. This proactive approach helps ensure that no cases of hearing loss go undetected, as it aims to identify hearing loss in all infants, including those who may not have obvious risk factors for hearing impairment (Kanji, 2021).

The World Health Organization (WHO, 2021a, 2021b) proposes UNHS as the preferred screening approach. A recent systematic review and meta-analysis confirmed positive outcomes associated with UNHS (UNHS Review Group, 2022). These outcomes include an increase in the proportion of infants diagnosed with permanent bilateral hearing loss by 9 months of age and a 13-month improvement in the mean age of hearing loss diagnosis (UNHS Review Group, 2022).

The Health Professions Council of South Africa (HPCSA) supports the UNHS initiative and recently published updated early hearing detection and intervention (EHDI) guidelines (HPCSA, 2018). The development of this contextualised guideline was guided by international best practice. Two platforms for NIHS in South Africa are proposed: hospital based (e.g. at discharge from well-baby nurseries or intensive-, high care and kangaroo mother care wards) and community based (e.g. primary health care [PHC] clinics and midwife obstetric units [MOUs]) (HPCSA, 2018). For community-based screening programmes linked to immunisation visits, the South African HPCSA principles state that initial screening should be conducted by 6 weeks of age, hearing loss should be confirmed by no later than 4 months of age and intervention provided before 8 months of age. These contextual timeframes for screening in South Africa differ from the international gold standard for EHDI programmes, the 1-3-6 principle (HPCSA, 2018). This principle recommends screening for hearing loss before 1 month of age, diagnostic evaluation before 3 months of age and enrolment in early intervention before 6 months of age (JCIH, 2019). The South Africa EHDI guidelines also outline benchmarks and quality indicators. The benchmarks for community-based screening programmes are that: (1) within 6 months of programme initiation 95% of infants attending their 6-week immunisation or postnatal follow-up visits should be screened; (2) within 1 year of programme initiation the referral rate for audiological and medical evaluation should be < 5% and (3) the follow-up return rate should be > 70%. Several quality indicators are provided, such as mean age at initial screening and percentage of families who refuse community-based hearing screening (HPCSA, 2018).

A recent study conducted in the province of KwaZulu Natal found that although EHDI guidelines are available, audiologists’ perceptions deemed the guidelines as more suitable for urban contexts (Naidoo & Khan, 2022). Even though the HPCSA published a Hearing Screening Position Statement in 2002, the first EHDI guidelines were published in 2007, and the guidelines were updated in 2018, community-based UNHS is still in its infancy. Several short-term screening projects were implemented in the metropolitan areas of the Western Cape Province at maternal and childcare clinics (Friderichs et al., 2012) and MOUs (De Kock et al., 2016). Two Gauteng-based studies were conducted: one at immunisation clinics (Swanepoel et al., 2006) and another at an MOU (Khoza-Shangase & Harbinson, 2015). The outcomes of these projects varied in terms of the achievement of key benchmarks and the challenges faced in sustaining the projects. Overall, the use of community-based platforms, specifically the 3- and 10-day postnatal follow visits (at MOUs and PHC clinics) and the 6-week immunisation visit (at PHC clinics), was found to be suitable for the South African urban context.

Despite the confirmation of the suitability of community sites (such as MOUs and PHC clinics), community-based UNHS has not been realised in South Africa. Lack of political will (Maluleke et al., 2024), and the subsequent lack of resource allocation (Naidoo & Khan, 2022; Petrocchi-Bartal et al., 2021) is cited as reasons. In addition to the lack of screening equipment, human resources remain a challenge. The unequal distribution of audiologists between the provinces and urban and rural contexts hampers the implementation of UNHS. The ratio of speech-language and hearing professionals (e.g. audiologists, speech-language therapists and dually registered audiologists and speech-language professionals) per 10 000 population is considerably higher in provinces with more urban households (e.g. Western Cape [1.03] and Gauteng [0.97]) compared to provinces with more rural households (e.g. Limpopo [0.22]) (Pillay et al., 2020).

The global shortage of audiologists has necessitated the implementation of task shifting. Task shifting is a process where specific tasks are redistributed, where appropriate, to individuals with fewer qualifications (Maier & Aiken, 2016). Several studies support and promote task shifting for ear and hearing care programmes (Eksteen et al., 2019; Suen et al., 2019). One such audiologist-managed programme offered by salaried community health workers (CHWs) is hearing and vision screening for preschool children. Through this programme, accessibility of the services for children in the community improved and referral rates to already overloaded public hospitals decreased (Eksteen et al., 2019). The aim of the current study was therefore to describe the outcomes of a 1-year UNHS pilot programme implemented at three PHC clinics in the Limpopo province.

## Research methods and design

### Setting

The Elias Motsoaledi Local Municipality is a mainly rural area with only one major town (Integrated Development Plan, 2021). Hearing health services are available at two facilities, a district hospital and a non-profit community-based audiology clinic. The district hospital (situated between 1 km and 18 km from the PHC clinics) offers basic diagnostic assessments and management of hearing loss. The community-based audiology clinic (situated between 5 km and 17 km from the PHC clinics) offers free diagnostic assessments and management of hearing loss. This is the only facility in the area with diagnostic auditory brainstem response (ABR) equipment.

The UNHS programme was established in July 2014 at three of the government-run PHC clinics in the area by a non-profit organisation. These PHC clinics provide comprehensive nurse-led services including care for chronic illnesses, maternal and childcare, family planning and immunisations. Hearing screening services were provided by trained hearing screeners (under the supervision of two HPCSA registered audiologists) and were available Monday to Friday (07:00 to 13:00).

### Screening personnel

Three community members, each with a matric certificate and basic computer literacy, were trained by the audiologist employed by the non-profit organisation. The 10-day training included theoretical and practical sessions and covered various topics. The theoretical component included the principles and rationale for EHDI, basic overview of the anatomy and physiology of the ear, developmental milestones (hearing, speech and language), screening measures and equipment, test protocol, and counselling. The practical training included the ability to screen, troubleshoot equipment challenges, conduct pre- and post-screen counselling, make recommendations and appropriate referrals, give hearing health information talks as well as administration (e.g., recordkeeping and data management) (HPCSA, 2007). Screeners were only deemed competent if they passed the theory component by more than 80% on a test and successfully completed 10 supervised screens. In the first 3 months of the programme, quality control and support visits took place on a weekly basis and changed to monthly in the last 9 months of the programme.

### Equipment

The screening was conducted with the MB 11 BERAphone^®^, an AABR system. It is a single unit with three integrated electrodes and a speaker with ear cushion. Electrode gel is applied on the infants’ head at the three electrode sites (vertex, mastoid and ground). A *pass* result is recorded if a response is detected and verified at 35 dBnHL (https://www.maico-diagnostics.com/products/abr/mb-11-beraphone). Automated auditory brainstem response assesses the auditory nervous system (Ngui et al., 2019). The negligible consumable costs, reduced preparation time, lower initial referral rates and higher true positive rates, when compared to otoacoustic emission (OAE) testing, make AABR screening a suitable option for community-based screening programmes (De Kock et al., 2016). The lower referral rates further reduce the costs associated with follow-up assessments (Stewart et al., 2000). In line with the HPCSA (2007) and JCIH (2007) guidelines, diagnostic assessments were conducted by the audiologists in either the community-based audiology clinic and the district hospital. These assessments included assessments of outer ear integrity (otoscopic examination), assessment of middle ear functioning using a high-frequency probe tones of 1000 Hz for infants younger than 6 months, acoustic reflex thresholds, ABR (neurodiagnostic and threshold estimation) as well as parental feedback on emerging communication and auditory behaviours (JCIH 2007). However, the district hospital was able to conduct only outer ear (otoscopic examination), middle ear functioning (226 Hz) assessments as well as the parental feedback on the communication and behavioural because of malfunction and shortage of equipment.

### Protocol

Every morning hearing screeners gave a hearing health talk in the clinic waiting area and invited caregivers to have their infants’ hearing screened. Interested caregivers were directed to the screening room.

A two-stage protocol, with a bilateral pass criterion, was adopted:

- Step 1: Explain the purpose of hearing screening and screening procedures.
- Step 2: Caregivers sign an informed consent form, which also includes consent to use results for research purposes.
- Step 3: Screeners complete a screening form with information on demographic information, brief family and medical case history, high-risk checklist obtained from the caregiver and screening outcomes for each screening session.
- Step 4: Perform AABR screening. The screener was not allowed to repeat the measurement unless the test was ‘incomplete’.
- Step 5: Record results on the screening form and in the Road to Health booklet.
- Step 6: Give verbal feedback to the caregiver as well as in the pamphlet on normal hearing-, speech- and language development in the caregivers’ preferred language:
- Step 6 A: Bilateral ‘Pass’ result – discharge and recommend annual monitoring.
- Step 6 B: Unilateral or bilateral ‘Refer’ result – Book second screen appointment to coincide with the next postnatal follow-up visit or 2 weeks from the initial screening date. Community Health Worker confirms appointment with the caregiver 2 days before the appointment. Perform AABR screening as in Steps 3, 4, 5 and 6.
- Step 6 C: Unilateral or bilateral ‘Refer’ result after second screen – record screening results as per protocol and refer for diagnostic assessment to the nearest audiology department (e.g. district hospital or non-profit audiology clinic and dependent on caregiver preference).

Only the community-based audiology clinic offered comprehensive diagnostic assessments that included otoscopy, tympanometry (226 Hz and 1000 Hz) and diagnostic ABR. The district hospital unfortunately only conducted otoscopy and tympanometry because of a lack of equipment.

When the hearing screening services were first implemented (first 6 months of the programme), all infants younger than 12 months whose parents were interested and consented to their infants’ hearing being screened were screened. This was on request of caregivers who were excited about the new service offered at the clinics. The screening age was reviewed after 6 months to align with the HPCSA guidelines for community-based hearing screening programmes of conducting hearing screening on or before 6 weeks (HPCSA 2007, 2018). Screening was then only offered to caregivers at the 3-day postnatal, 10-day postnatal and 6-week immunisation visits.

### Study design

The study employed a descriptive retrospective review of 2302 audiological records of infants of 0–12 months whose hearing was screened at three PHC clinics in Limpopo Province between July 2014 and June 2015.

### Data collection

Two data-extraction sheets were developed using Microsoft Excel. The development was guided by the 2007 HPCSA guidelines. The first sheet was used to extract data from individual screening records and the second to record PHC clinic statistics. The first sheet comprised information such as age (in days) at screening, risk factors, first screen outcomes (per ear), second screen results per ear (where relevant), further referrals and diagnostic outcomes. The second sheet comprised information on the number of infants attending visiting the clinic for the 3- and 10-day postnatal visits as well as the 6-week immunisation visit between July 2014 and June 2015. In addition, the number of infants screened for the first time while attending the clinic for 3- and 10-day for postnatal visits, 6-week immunisation visit and older than 6-week clinic visits as well as second screens were included. The second sheet focused on the number of infants whose hearing was screened while attending the 3- and 10-day postanal visits as well as the 6-week immunisation visit, and it was also used to record the clinic statistics of the number of infants that visited the clinic for the 3- and 10-day postnatal visit and 6-week immunisation.

Only the records of infants whose caregivers gave written consent for the screening results to be used for research purposes were included in the study. Records with incomplete hearing screening results or missing data were excluded from the study.

### Data analysis

All data were captured in Microsoft Excel 2016 by the main author (audiologist) who also verified the data for accuracy. Descriptive statistics were used to calculate the mean age at screening, coverage rate, as well as referral- and follow-up rates. These rates were calculated as follows:

#### Coverage rate

Number of infants initially screened at the PHC clinics compared to the total number of infants who attended clinic for 3- or 10-day postnatal visit and the 6-, 10- or 14-week immunisations.

#### Referral rate

This was calculated for the first- and second-stage screening using the number of infants with a refer result (unilateral or bilateral) compared to the total number of infants screened.

#### Follow-up rate

Follow-up rate was reported for follow-up screen (at clinic level) as well as follow-up for diagnostic assessment (at the hospital level).

### Ethical considerations

Ethical clearance was obtained from the Human Research Ethics Committee (Medical) of the University of the Witwatersrand before the commencement of the study (Protocol number: M150958).

As part of screening procedures, caregivers were requested to sign a consent form that permits the programme to use the results for research purposes. For this study, only records where caregivers gave written consent to use the audiological records for research purposes were included. Then, written permission to review the records was obtained from the chief executive officer of the NGO who offered the newborn hearing screening services at the PHC clinics. Records were allocated participant numbers, and to maintain anonymity, no identifying information was recorded.

## Results

A total of 2378 records were reviewed. Of these, 76 were excluded because of incomplete forms (*n* = 15) and lack of consent to use results for research purposes (*n* = 61). A total of 2302 records were included for analysis.

### Mean age at initial screening

The overall mean age at initial screening for the review period was 16 weeks (112 days) (Table 1). The average age for the first 6 months of programme implementation was 24 weeks (168 days), which decreased to 8 weeks (56.88 days) in the last 6 months of the programme. In this last period, screening was only offered to caregivers at the 3-day postnatal, 10-day postnatal and immunisation visits at 6, 10 and 14 weeks.

**TABLE 1:** Mean age of screening (in days).

Age (days)	July 2014 – December 2014 (*n*_1_ = 1350)	January 2015 – June 2015 (*n*_2_ = 952)	Total (*N* = 2302)
Mean	168	56.88	112
Range	2–293	2–187	2–341
Standard deviation	54.35	40.56	40.26

**FIGURE 1:**
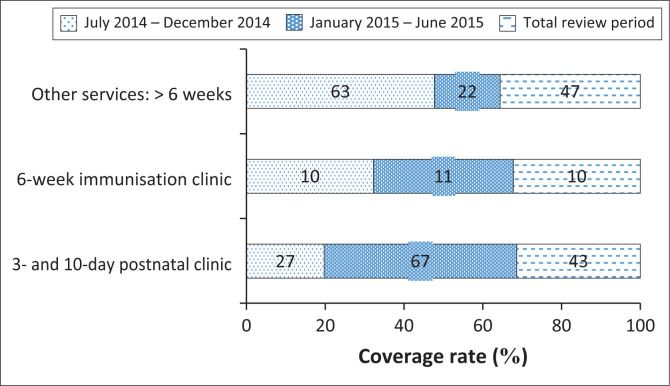
Hearing screening coverage rate.

In the last 6 months of the programme, screening was only offered to caregivers at the 3-day postnatal, 10-day postnatal and immunisation visits (6, 10 and 14 weeks) (Figure 1). This change ensured that the programme was closely aligned to the HPCSA guidelines. Most infants screened (67%) in this period were from the 3- and 10-day postnatal clinic.

Clinic statistics were only available for the 3- and 10-day post-natal visit and the 6-week immunisation visits. Table 2 provides information on the number of infants screened compared to the number of infants attending the clinic for post-natal and immunisation visits.

**TABLE 2:** Coverage rate.

Visits	No. infants screened	Total no. infants attending clinic service	Coverage rate (%)
3- and 10-day post-natal visit	1001	1148	87
6-week immunisation visit	239	1417	27
Older than 6 weeks	1062	Information not provided by clinic	-

Note: Coverage rate = infants screened/infants attending clinic.

### Screening outcomes

**FIGURE 2:**
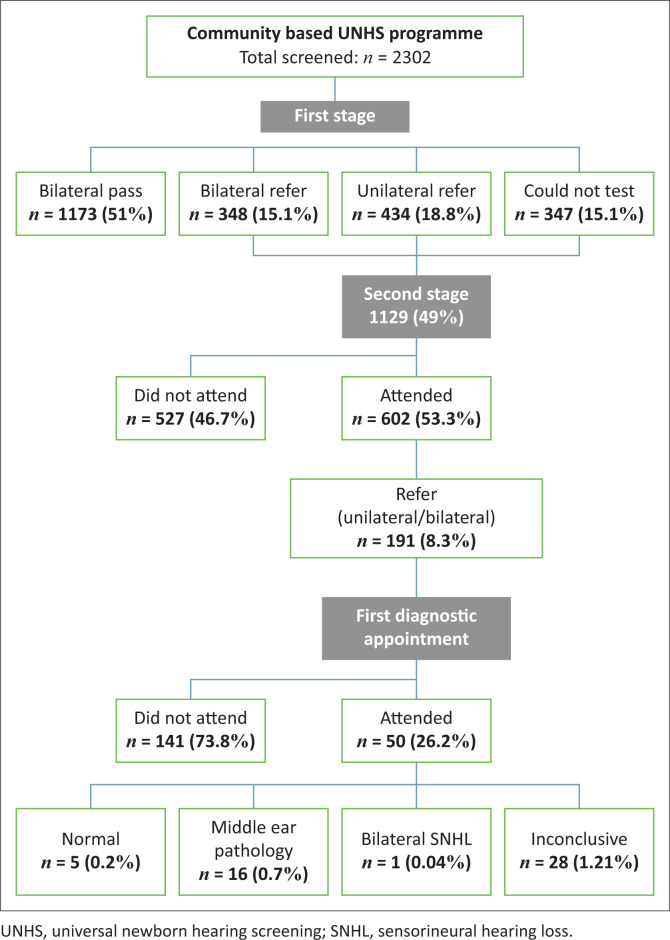
Schematic presentation of screening outcomes of community-based universal neonatal hearing screening service.

A summary of the screening outcomes (stage 1, stage 2 and first diagnostic appointment) is presented in Figure 2.

#### Coverage rate

Clinic statistics were only available for the 3- and 10-day post-natal visit and the 6-week immunisation visits. Table 2 provides information on the coverage rate of the screening programme. The coverage rate for the 3- and 10-day post-natal visit was 87% and for the 6-week immunisation visit, only 27%.

#### Referral rate

Overall, the referral rate for first-stage screen was 33.9% (782/2302). A total of 18.8% (434/2302) of the infants were referred for one ear while 15.1% (348/2303) were referred for both ears. A further 15.1% (*n* = 347) of the infants could not be tested because of infant state (restlessness or irritability.) The referral rate for the second stage screen was 8.3% (191/2302). Infants who referred during this stage were referred for diagnostic audiology services (district hospital or community-based audiology clinic) depending on their caregiver preference.

#### Follow-up rate

As is evident from Figure 2, the follow-up rate at the clinic level was 53% (602/1129). The follow-up rate for those who were referred for a diagnostic assessment was 26% (50/191).

#### Outcomes: First diagnostic appointment

A total of 50 infants returned for a first diagnostic assessment at either the district hospital (*n* = 27) or the community-based audiology clinic (*n* = 23). A bilateral sensorineural hearing loss was confirmed in one infant who was subsequently referred to an academic hospital for further management. Five infants presented with normal hearing. Middle ear pathology (e.g. middle ear effusion) was identified in 16 infants who were referred for medical management. In addition, the results for 28 infants were inconclusive. Inconclusive results were because of restlessness during testing (*n* = 6) and lack of equipment for diagnostic assessment at the district hospital (*n* = 22). The prevalence rate for bilateral sensorineural hearing loss was 0.4/1000 (1/2302), and middle ear pathology was 6.9/1000 (16/2302).

## Discussion

The outcomes of this rural community-based screening programme were evaluated against benchmarks and indicators outlined in the EHDI Guidelines: Year 2018 (HPCSA, 2018).

### Coverage rate

The overall coverage rate of 87% for 3- and 10-day post-natal follow-up visits for this rural community-based screening is lower than the > 95% coverage rate suggested by EHDI Guidelines: Year 2018 (HPCSA, 2018). Additionally, a 27% coverage rate was achieved for the 6-week immunisation visits in comparison to the 95% coverage rate stipulated by the HPCSA (2018). The high coverage rate for the 3- and 10-day infants (87%) and the lower coverage rate for the 6-weeks old infants (27%) could be because most infants attending the 6-week immunisation clinic were screened at their 3- or 10-day postnatal visits. Although the coverage rate in our study is lower compared to the rate suggested by HPCSA, it supports research that confirms that screening linked to these visits has the potential to become the most effective community-based platform for screening in South Africa (De Kock et al., 2016; Friderichs et al., 2012; Khoza-Shangase & Harbinson, 2015). The coverage rate reported in the current study is significantly higher than rates reported for other South African NIHS programmes (De Kock et al., 2016; Friderichs et al., 2012). In the Western Cape, an extensive community-based UNHS programme for 0- to 14-week-old infants reported a coverage rate of 32.4% over a 19-month period (Friderichs et al., 2012). In the current study, hearing screening was conducted by dedicated hearing screeners unlike in the aforementioned studies that mainly relied on nursing personnel to conduct the hearing screening in addition to their other responsibilities (Friderichs et al., 2012). A private hospital-based NHS programme in Gauteng reported a 75% coverage rate over a 22-month period while the service was included in the hospital birthing package, which decreased to 20% in the last 26 months when it was not part of the birthing package (Swanepoel et al., 2007). A recent report on the global status of NIHS indicated that only 33% of countries who participated in the survey achieved a coverage rate of above 85% (Neumann et al., 2020). For the current study, the high coverage rate for the 3- and 10-day post-natal visits can be attributed to the fact that parents were well informed of the objectives of this free screening service and that screening was conducted by dedicated hearing screeners.

### Referral rate

The current study reported a higher referral rate (8.3%) in comparison to the national (< 5%) guidelines. The referral rate is similar to rates reported in other low- and middle-income countries such as Brazil (6.8%) and India (6%) (Béria et al., 2007). The referral rates reported for South African programmes varied significantly, depending on the screening protocol used. Programmes that conducted AABR screening reported an initial referral rate of 4.6%, which decreased to 0.3% after the second stage (De Kock et al., 2016). The referral rates for programmes using OAE screening (transient evoked OAE or distortion product OAE) were higher in community-based (De Kock et al., 2016) as well as hospital-based screening (Friderichs et al., 2012; Van Dyk et al., 2015) when compared to AABR screening. For the current study, the contributors to the high referral rate may be the state of alertness of the infant. Considering the mean age at the initial screening of 112 days, the infants’ alertness may have affected the outcome or reliability of the test results because of the myogenic interference (Olusanya, 2015). Research confirmed a higher referral rate for older infants (Chung et al., 2019).

### Follow-up rate

High loss-to-follow up rates remain a challenge internationally. A recent global survey found that 48% of hearing screening programmes failed to meet the > 70% follow-up rate (Neumann et al., 2022). The current study achieved mixed successes, with a 53% follow-up rate at the clinic level but a 26% follow-up rate for infants referred for diagnostic assessments. Other NIHS programme in South Africa reported average follow-up return rates of 80% at clinics in the Western Cape (Friderichs et al., 2012), between 86.6% and 92.3% at MOUs in the Western Cape (De Kock et al., 2016), between 40% and 44% at clinics in Gauteng (Swanepoel et al., 2006) and between 35.8% and 56.1% for hospital-based screening (Scheepers et al., 2014). Considering the rural context of the current study, as well as the high unemployment rate, reasons for the low follow-up rate for infants referred for diagnostic assessment could be ascribed to distance to the district hospital or community-based audiology clinic and transportation challenges and costs for attending the follow-up services (Mackey at al., 2022).

### Mean age at initial screening

The overall mean age at initial screening in the current study (112 days; 16 weeks) was much higher than the age outlined in the EHDI Guidelines: Year 2018 (HPCSA, 2018) for clinic-based programmes, that is 6 weeks. A 6-month review of the programme and as the community became more aware of the programme during the last 6 months of the programme led to a decrease in the mean age of infants screened (8 weeks). Despite this, the mean age in the last 6 months was higher (56 days, 8 weeks) than results reported for hearing screening conducted at MOUs (6.1 days) (De Kock et al., 2016) and immunisation visits (3.9 weeks) (Friderichs et al., 2012) in the Western Cape. The importance of regular monitoring of screening programme and strict adherence to the guidelines was confirmed in the current study.

### Diagnostic outcomes

The prevalence rate for bilateral sensorineural hearing loss, at 0.4/1000, is comparable to prevalence rates ranging from 0.3 to 15 per 1000 infants (median of 1.7) obtained from a global survey (Neumann et al., 2022). Similar prevalence rates were reported for community-based screening programmes in South Africa. Reported prevalence rates were 0.3/1000 for infants screened with DPOAE (De Kock et al., 2016), 1/1000 for infants screened with AABR (De Kock et al., 2016) and 1.5/1000 screened with DPOAE at PHC clinics in the Western Cape (Friderichs et al., 2012). An estimated prevalence rate of 3/1000 was reported for a hospital-based screening programme (Swanepoel et al., 2007). The prevalence rate for middle ear pathology for the current study of 6.9/1000 was lower than the rates reported in the Western Cape at 12.9/1000 (Friderichs et al., 2012). The prevalence of middle ear pathology in children aged 0–3 years in the same area where the current study was conducted was 1.05% and 2.48% of the general population (Joubert & Botha, 2019). As middle ear pathologies, such as middle ear effusion, may mask sensorineural hearing loss, it is important to closely monitor these cases (Friderichs et al., 2012).

### Implications

This is the first study conducted in South Africa to determine the feasibility of community-based UNHS services in rural areas. These findings have implications for policy and programme implementation as they prove that community-based UNHS is feasible in South Africa. Future research should endeavour to evaluate NIHS programmes more holistically by also exploring the perspectives of health care workers and screening personnel on the effectiveness of the NIHS programmes. The study can be replicated and expanded over a 5-year period from programme implementation to evaluate its effectiveness and determine whether there was any improvement in meeting the national and international benchmarks.

## Conclusion and recommendations

The study’s findings demonstrate the feasibility of implementing clinic-based NHS programmes in resourced-constrained community settings. Although not all the benchmarks were attained within the first year of implementation, the high coverage- and low referral rates are the first steps in improving the outcomes of the screening programme. The decrease in the mean age at initial screening in the last 6 months of the review period highlights the need for and positive impact of regular monitoring and evaluation of NIHS programmes (HPCSA, 2018). The high coverage rate validates the much-discussed topic of adopting task shifting as a public health initiative, that is training CHWs or community members to conduct hearing screening, to address the glaring gap in audiology human resources (Pillay et al., 2020; WHO, 2021a, 2021b). Lastly, the results indicate the positive impact of public–private partnerships on the facilitation of access to healthcare services, including NIHS, in rural settings (Joudyian et al., 2021). This should be an important consideration to facilitate the achievement of the WHO’s 2030 goal of increasing the NIHS coverage rate by 20% (WHO, 2021a).
